# Exploring how the ambidextrous leadership influences knowledge workers innovative behavior: a two stage SEM-ANN analysis

**DOI:** 10.3389/fpsyg.2025.1560726

**Published:** 2025-06-06

**Authors:** Tingxiu Guo, Dengkai Zhang, Juanli Yang, Jintao Xia

**Affiliations:** ^1^School of Finance and Economics, Qinghai University, Xining, Qinghai, China; ^2^Shanghai Jianqiao University, Shanghai, China

**Keywords:** ambidextrous leadership, knowledge workers, innovative behavior, perceived organizational support, self-efficacy, SEM-ANN

## Abstract

This study investigates the impact of ambidextrous leadership, which integrates transformational and transactional leadership styles, on the innovative behavior of knowledge workers. Grounded in theory of reciprocal determinism, it explores the mediating roles of perceived organizational support and self-efficacy, addressing gaps in the literature on leadership and innovation. Data were collected from 372 knowledge workers in China via an online survey, and analyzed using a two-stage methodology that combines Partial Least Squares Structural Equation Modeling (PLS-SEM) to assess linear relationships and Artificial Neural Network (ANN) analysis to capture non-linear dynamics. The results indicate that ambidextrous leadership significantly enhances knowledge workers' innovative behavior through the mediating effects of perceived organizational support and self-efficacy, with a chain mediation effect underscoring the interplay between leadership, organizational support, and individual psychology. By integrating ambidextrous leadership with reciprocal determinism theory, this study enriches the theoretical understanding of leadership's role in fostering innovation and provides cross-cultural evidence of its applicability. The findings offer practical strategies for organizations to foster innovation by creating supportive environments and adopting adaptive leadership practices. Future research could explore longitudinal effects and investigate additional mediating or moderating variables to further deepen the understanding of leadership's impact on innovation.

## 1 Introduction

In a rapidly evolving and highly competitive market environment, innovation is a key determinant of an enterprise's survival and prosperity. As the environment grows increasingly dynamic and competitive, innovation decision-making becomes more complex. Innovation involves continually transcending existing knowledge, achieved through exploration and development activities. However, factors such as a team leader's energy, available resources, employee innovation capability, and organizational context limit these activities. Consequently, the relationship between these innovation activities and team performance is not linear but exhibits an inverted U-shape, overemphasis on one activity can diminish innovation performance (Sun and Yang, 2017). Addressing contradictions in innovation demands more from managers and has drawn scholarly attention to contradiction management. To better resolve these contradictions and meet organizational and employee needs, scholars recommend that leaders adapt their behaviors based on specific contradictions, shifting from unitary to dual thinking (Luo et al., 2016a). Knowledge workers are the primary drivers of innovation within enterprises, introducing new products, services, and work methods that enhance market competitiveness and adaptability (Ouyang et al., 2022). As global economic integration deepens and information technology becomes increasingly significant, knowledge resources have emerged as an enterprise's most valuable asset. Knowledge workers are central to enterprise innovation, already have attracted significant attention from both academia and the business sector (Abubakar et al., 2019). These workers typically possess advanced education and professional skills, enabling them to engage in creative thinking and problem-solving (Luo et al., 2016a). Thus, fostering the innovative behavior of knowledge workers is crucial for enterprises aiming to achieve sustainable competitive advantage and long-term growth.

Leadership plays a pivotal role in shaping employee innovation, as evidenced by decades of research. Transformational leadership fuels creativity through inspiration and idealized influence (Wei et al., 2021; Yousef, 2000), while transactional leadership provides structure via contingent rewards and exception management (Zhang, 2014). However, this binary categorization oversimplifies organizational realities. We argue that an either/or approach creates systemic risks: overemphasis on transformational styles may neglect operational discipline, whereas excessive transactional control can suppress exploratory thinking (Chen and Zhou, 2009; Wang et al., 2006). This tension demands ambidextrous leadership—a dynamic fusion of styles that addresses the innovation paradox.

Building on this paradoxical synergy, ambidextrous leadership represents a sophisticated approach that combines two seemingly contradictory but mutually reinforcing leadership tactics, signaling a new phase in leadership practice (Luo et al., 2016b). This paradigm advocates for an inclusive “both-and” perspective rather than a limiting “either-or” mindset, allowing leaders to flexibly adjust their strategies in response to environmental changes. Such adaptability is essential for fostering a synergistic balance and driving joint progress between initially opposing forces (Schreuders and Legesse, 2012).

In order to operationalize this “both - and” paradigm, ambidextrous leadership is manifested through three constitutive dimensions, namely cognitive, behavioral, and power. Each of these dimensions is specifically aimed at addressing the inherent tensions in innovation management. From a Cognitive Perspective, Rosing et al. (2011) propose a framework contrasting open and closed leadership behaviors. Open leadership promotes breaking conventional norms and fostering innovation, igniting the emergence of novel ideas. In contrast, closed leadership focuses on formalizing and enforcing protocols to ensure the effective implementation of these innovations. From a Practice Perspective, Schreuders and Legesse (2012) suggest combining transformational and transactional leadership strategies. Transformational leadership enhances organizational adaptability through motivational incentives and empowerment, while transactional leadership ensures operational consistency through the application of clear rules and regulations. Lastly, from a Power Perspective, Sagie et al. (2002) introduce the concept that the integration of empowering leadership, characterized by the delegation of authority, and commanding leadership, defined by its directive nature, forms the core of ambidextrous leadership. The integration of transformational and transactional leadership within ambidextrous leadership arises from their complementary nature. Transformational leadership promotes exploratory innovation through visionary inspiration (Bass, 1990), while transactional leadership provides stability. This dual capability aligns with Schreuders and Legesse (2012) has point out “both-and” paradigm, facilitating dynamic adaptation to environmental complexity.

While the positive effects of various leadership styles on employee satisfaction, loyalty, and innovative behavior have been extensively studied, there remains a notable gap regarding the concept of “ambidextrous leadership” and its mechanism in influencing the innovative behavior of knowledge workers through perceived organizational support and general self-efficacy. While existing studies, such as Luo et al. (2016b) outline the characteristics of ambidextrous leadership and its impact on organizational dynamics but lack of systematically exploring how ambidextrous leadership integrates with transformational and transactional leadership styles to influence the innovative behavior of knowledge workers. There are also large of research has focused on exploring the positive impacts of ambidextrous leadership on employee innovation and creativity (Han et al., 2016; Jia et al., 2024; Li, 2019; Luo et al., 2016a; Wang, 2020). In addition, several studies have highlighted the positive relationship between ambidextrous leadership and various employee outcomes, including job satisfaction, emotional engagement, professional development, and organizational efficiency (Lian et al., 2022; She and Tan, 2016; Sun et al., 2017; Sun and Song, 2015; Zhao and Guo, 2017). Research on this topic often lacks a cross-cultural comparative perspective, which limits our understanding of the universality of these leadership styles. Existing studies also exhibit several limitations: most focus predominantly on specific outcomes, such as creativity, neglecting broader employee characteristics, and fail to systematically investigate the innovation propensity among knowledge workers.

To advance ambidextrous leadership theory, this study applies Bandura's reciprocal determinism as an explanatory lens, particularly modeling how leaders' dual behaviors (transformational/transactional) interact with employees' cognitive states and environmental conditions in adaptive reciprocity cycles. This approach extends prior static models by introducing temporal dynamism to leadership ambidexterity. This integration provides a more nuanced understanding of how the balance between transformational and transactional leadership styles influences innovation, particularly through the mediating roles of perceived organizational support and self-efficacy. By focusing on knowledge workers in China, the study also extends the applicability of ambidextrous leadership to a cross-cultural context, shedding light on both its universal and culturally specific effects. To further explore these dynamics, the study poses the following research questions: how does ambidextrous leadership impact the innovative behavior of knowledge workers? What role does perceived organizational support and self-efficacy play in mediating the relationship between ambidextrous leadership and innovation? These questions guide the exploration of the complex mechanisms through which leadership fosters innovation in diverse organizational settings.

This study offers three pivotal contributions. Firstly, it identifies adaptive reciprocity loops as a key mechanism for ambidextrous leadership in addressing the innovation paradox. Secondly, it introduces a dual-analysis paradigm to encapsulate both linear and non-linear leadership dynamics. Methodologically, our research pioneers a two-stage analytical approach that synergizes Partial Least Squares Structural Equation Modeling (PLS-SEM) and Artificial Neural Networks (ANN). The former rigorously tests hypothesized linear relationships, while the latter detects hidden non-linear interactions and threshold effects inherent in ambidextrous leadership dynamics. This dual methodology transcends conventional approaches by harmonizing theory-driven hypothesis testing with data-driven pattern discovery a critical advancement given the paradoxical nature of leadership ambidexterity. Lastly, it elucidates the specific expressions of ambidextrous leadership within a knowledge-intensive context in China. The findings provide not only fresh perspectives for theoretical development but also practical recommendations for organizations, particularly regarding the optimization of leadership styles and the enhancement of employees' innovation.

## 2 Literature review and hypothesis development

### 2.1 Reciprocal determinism theory

Bandura (1978b) theory of reciprocal determinism is a seminal framework in psychology, emphasizing the dynamic interaction between personal agency, the surrounding environment, and individual actions. The central premise is that human behavior is not merely a response to external stimuli but is shaped by internal cognitive processes, including expectations, beliefs, and perspectives (Garrido, 2023). Bandura argues that learning occurs indirectly through observing others' behaviors and the outcomes they generate, a process known as observational or imitative learning.

The theory of reciprocal determinism has been widely applied across various psychological disciplines, including education, health psychology, and organizational behavior. It serves as a powerful tool for explaining how individuals learn and grow in social contexts, emphasizing the importance of personal initiative and creativity in driving behavioral change (Nickerson, 2024). This theory facilitates a deeper understanding of human behavior and provides valuable insights into promoting positive behavioral changes through the modification of individual beliefs and expectations. According to the principles of reciprocal determinism, individual actions, cognitive processes, and the external environment are intricately interconnected. This perspective challenges the limitations of traditional behaviorism and emphasizes the crucial role of cognitive processes in regulating behavior (Yin, 2022).

When examining the influence of ambidextrous leadership on the innovative behavior of knowledge workers, Bandura's theory highlights the central role of self-efficacy, which refers to an individual's belief in their ability to complete a specific task. Self-efficacy is a key factor influencing behavior and motivation. Hao (2024) suggests that self-efficacy can be developed through four primary pathways: personal mastery experiences, observation of others, social persuasion, and emotional state. Moreover, the perception of organizational support is recognized as a significant determinant of employee behavior. When employees perceive that their organization supports them, they are more likely to internalize organizational goals, which in turn enhances their engagement and innovation (Imran et al., 2020).

**Figure 1:**
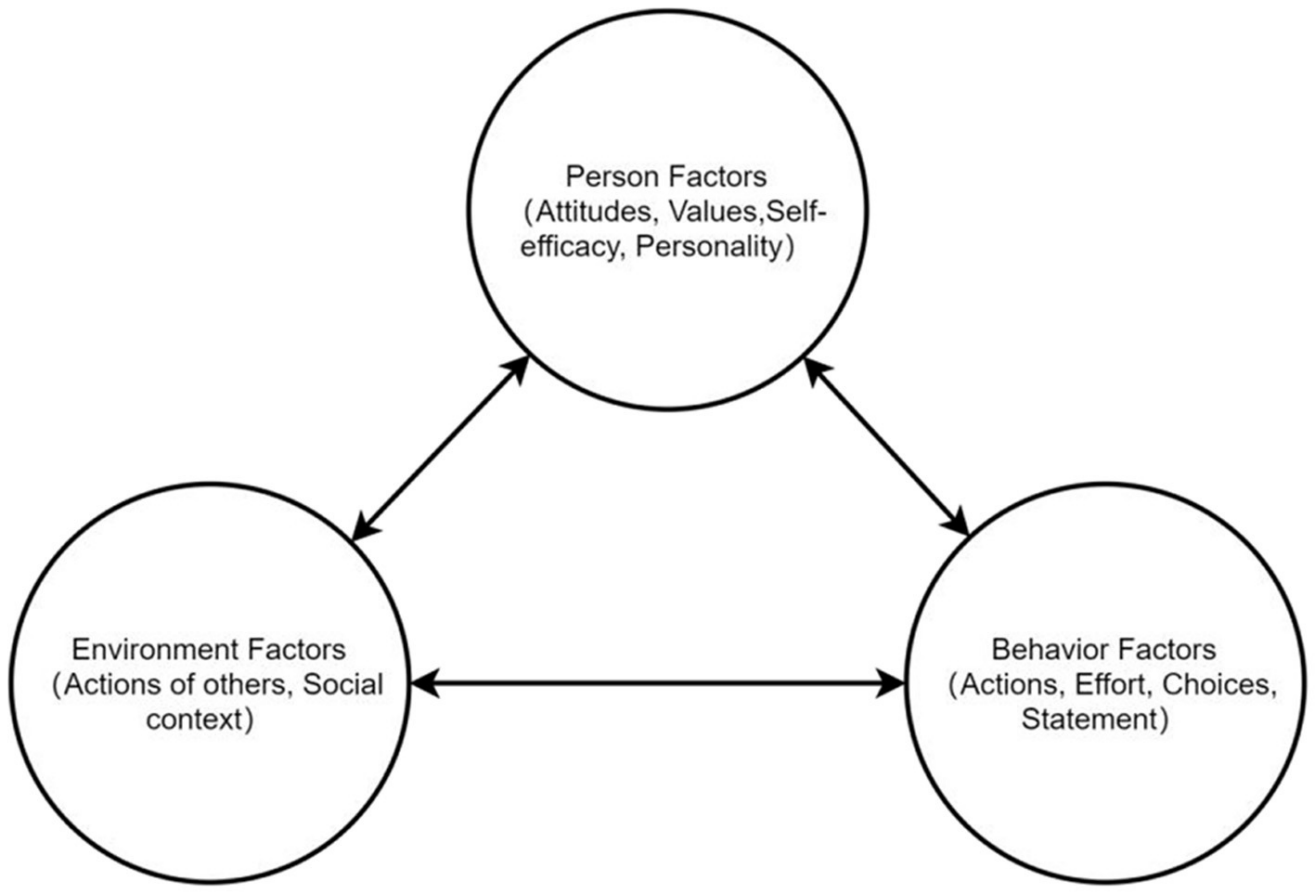
Based on Bandura (1978a).

Ambidexterity leadership is rooted in social exchange theory, fosters a mutual obligation dynamic (Cropanzano and Mitchell, 2005). Transformational behaviors develop socio-emotional resources via individualized consideration and intellectual incentives (Abbas et al., 2014), while transactional behaviors facilitate the exchange of economic resources through contingent rewards and performance monitoring (Meinecke et al., 2017). This dual-channel approach aligns with Zacher and Rosing (2015) ambidexterity paradox framework, where complementary leadership practices synergistically enhance perceived organizational support (as shown in Figure 1).

According to this theory, our research refines the concepts of “environmental factors” and “individual factors” by introducing “perceived organizational support” and “self-efficacy” as mediating variables, thus demonstrating their bridging role between ambidextrous leadership and the innovative behaviors of knowledge-based employees. By including ambidextrous leadership in the model—considered one of the external factors influencing employee innovation—the study underscores the leader's capacity to balance exploratory and exploitative innovations during organizational change.

### 2.2 Hypothesis development

Sun (2021) highlighted that an innovative organizational culture is a key external environmental factor that influences employees' innovative behavior. In particular, a positive organizational culture can stimulate employees' enthusiasm for innovation and promote individual innovative activities. By cultivating an atmosphere that supports innovation and tolerates failure, companies can effectively enhance employees' innovative behavior (Dai et al., 2024). Building on this, Li (2016) further suggested that work values significantly influence the innovative actions of individuals engaged in knowledge-intensive work. Specifically, employee values, such as the pursuit of comfort and safety, competence and growth, status, and independence, directly affect their innovative performance. Therefore, it can be reasonably inferred that employees who prioritize growth and competence are more likely to engage in innovative behavior at work.

Behavior modification within organizations represents a structured methodology designed to cultivate and reinforce desired behaviors through a series of deliberate, incremental tactics (Stajkovic and Luthans, 1997). This approach embodies the practical application of reinforcement principles within organizational governance. The importance of behavior modification is particularly evident in management contexts where a notable disparity exists between employees' behavioral performance and the aspirations and objectives of management (Miner, 2015). In such cases, it may be unrealistic to expect employees to autonomously adopt the desired behaviors. If incentives are provided only upon the achievement of predetermined benchmarks, they may seem too distant to effectively motivate employees. Therefore, the achievement of organizational goals can be significantly enhanced through behavior modification strategies that proactively and systematically guide employees in the gradual development of targeted behaviors.

By employing behavior modification techniques, including the strategic use of incentives and disincentives, organizations can effectively shape and guide employees toward adopting targeted behaviors. Proactive leadership in fostering an innovation-conducive environment can significantly reduce the uncertainties and risks inherent in the process, thus motivating employees to pursue innovative solutions with greater enthusiasm (Peng et al., 2020). From a practical perspective, transformational leadership is crucial in enhancing an organization's adaptability and agility through motivational strategies and individual empowerment. In contrast, transactional leadership ensures stability and the ongoing functionality of routine activities by adhering to well-defined protocols and guidelines (Liu et al., 2024). The meta-analytic findings of Judge and Piccolo (2004) demonstrated a medium-sized average correlation between transformational leadership and innovation outcomes (ρ = 0.44, *k* = 93 studies). Conversely, transactional leadership exhibited a significant yet smaller effect (ρ = 0.39, *k* = 91 studies). This suggests that while both leadership styles contribute to innovation, transformational leadership may be more strongly associated with such outcomes. Furthermore, Ng (2017) encompassing over 600 studies revealed that transactional leadership is less effective within explicit task contexts, with a 95% confidence interval of 0.24–0.34, indicating its potential as a complementary mechanism to transformational approaches. Based on the theoretical perspectives and empirical studies outlined above, this study proposes the following hypotheses:

Hypothesis 1: ambidextrous leadership has a significant positive effect on Knowledge Worker innovation behavior.
Hypothesis 2: perceived Organizational support has a significant positive effect on Knowledge Worker innovation behavior.
Hypothesis 3: general Self-efficacy has a significant positive effect on Knowledge Worker innovation behavior.

A substantial body of research on organizational support has consistently demonstrated its influence on various aspects of employees' professional lives, including their attitudes, behaviors, and overall organizational performance (Sun, 2019). Numerous studies have established a positive correlation between organizational support and self-efficacy. Specifically, a supportive organizational environment has been shown to enhance employees' self-efficacy (Kurtessis et al., 2017). This relationship also extends to emotional regulation, where organizational support has been significantly and positively associated with employees' ability to manage their emotions (Wei and Mao, 2018). Furthermore, the impact of organizational support on creative efficacy is significant, with studies highlighting a strong positive effect on employees' capacity for innovation (Gu and Zhou, 2014). Based on these findings, this study proposes the following hypothesis:

Hypothesis 4: perceived organizational support has a significant positive effect on employees' general self-efficacy.

The concept of organizational support is not inherent; conversely, it is a foundational element in shaping employees' perceptions of their employer's concern. The theory suggests that employees' recognition of the support they receive is critical and closely tied to their commitment to the organization. Eisenberger et al. (1986) demonstrated that transformational leaders can significantly influence job performance through prompt communication, motivation, attention to employee needs, performance feedback, and clear articulation of organizational policies. This influence is closely linked to employees' perceptions of the support provided by the organization, which can reinforce their commitment.

Yousef (2000) emphasized that transformational leadership not only enhances job performance but also drives the strategic growth of the organization by fostering a supportive environment for employees. In this context, organizational support plays a critical role in strengthening the correlation between transformational leadership and employee behavior. Transactional leadership, through the establishment of clear task expectations, role definitions, performance metrics, and the provision of contingent rewards, creates a transactional interaction. As evidenced by the study conducted by Fu and Ding (2016), the deliberate implementation of transactional leadership can effectively enhance employees' perceptions of organizational support. Social exchange theory posits that specific leadership styles can meet employees' support needs, thereby enhancing their perception of organizational support. Aligning support with employee needs facilitates this perception; the greater and more comprehensive the support, the stronger and more complete the perception of support. High levels of perceived organizational support can motivate employees to reciprocate, fostering a positive feedback mechanism that can improve organizational effectiveness.

Mayfield and Mayfield (2012) defined transformational leadership as an approach that promotes ongoing learning and personal development among organizational members, thereby enhancing their confidence in their abilities. By articulating a shared vision, transformational leaders motivate employees to proactively pursue objectives while fostering confidence and readiness for change. Furthermore, effective communication from leaders in the areas of feedback, goal setting, and training is crucial for improving employees' readiness to embrace change. Bass (1990) proposed that transactional leadership facilitates performance improvement by providing oversight and guidance, promptly addressing deviations from established procedures, and rewarding task completion. Although transactional leadership may lack the inspirational qualities of transformational leadership, it can still motivate employees to work diligently in pursuit of both tangible and intangible rewards, as noted by Lowe et al. (1996). Conger and Kanungo (1988) confirmed the positive effect of transactional leadership on self-efficacy in their empirical study. This leadership style was observed to foster employees' self-efficacy, drive them to work diligently, and strengthen their confidence and capability in task completion. Arnold et al. (2000) also found that transactional leadership significantly enhances employees' self-efficacy and contributes to a more congenial and relaxed work environment. Based on the established understanding of ambidextrous leadership behaviors, the following hypotheses are formulated:

Hypothesis 5: ambidextrous leadership has a significant positive effect on Perceived organizational support.
Hypothesis 6: ambidextrous leadership has a significant positive effect on employees' general self-efficacy.

The positive relationship between employees' recognition of an organization's support, both material and emotional, and their loyalty and constructive actions is well-established. Organizations, following the principle of reciprocity, seek to unlock the potential of their workforce through incentives, expecting meaningful contributions in return. However, it is important to recognize that employees' responses are not static; they adapt their behaviors based on their perception of organizational support (Lou, 2023). This indicates that organizational support serves as a critical mediator in the employer-employee relationship, significantly influencing staff conduct and attitudes.

In the existing literature, perceived organizational support is widely recognized as an essential intermediary. Ma and Liu (2020) emphasize that innovative leaders, as custodians of corporate culture, play a crucial role in shaping employees' perceptions of support for innovation by actively promoting and acknowledging their innovative efforts. Employees interpret the organization's expectations through its systems and the behaviors of its leaders, striving to align their actions with these expectations.

Rhoades and Eisenberger (2002) underscore the importance of leadership in shaping employees' perceptions of organizational support, including the quality of leader-member relationships, the level of leader support, and the prevailing leadership style. Notably, support from higher-status superiors is directly and positively linked to employees' perceptions of organizational support. Greater support results in a stronger perception of organizational backing, which can directly motivate individuals to engage in behaviors that benefit the organization, such as identifying with the organization, offering suggestions, and demonstrating organizational citizenship.

As organizations increasingly prioritize innovation, researchers have explored the link between perceived organizational support and employees' innovative behaviors. Yang et al. (2021) found that the care dimension of perceived organizational support can directly influence an individual's propensity for innovation. Moreover, perceived organizational support may also have indirect effects on various outcomes through mediating variables, a model widely applied in research to understand its impact on performance, turnover intentions, employee behaviors, and job satisfaction (Hao and Yang, 2023).

Self-efficacy, defined as the conviction in one's ability to successfully execute a task using acquired skills, occupies a central place in psychological studies (Bandura, 1978a). There is a significant positive correlation between self-efficacy and employees' innovative behavior, with those possessing higher self-efficacy typically exhibiting a stronger drive for achievement and a greater willingness to initiate and carry out innovative strategies. Their resilience in facing challenges further fuels their innovative activities. Xie (2018) highlighted the importance of examining how job characteristics affect employee adaptability, especially in relation to psychological traits like self-efficacy. Specifically, under transformational leadership, employees with higher self-efficacy are more likely to feel motivated to take on responsibilities and effectively utilize their work environment to achieve personal objectives. Luo et al. (2016a) demonstrated that self-efficacy mediates the link between Ambidextrous leadership behavior and employee behavior. The study revealed that dual leadership significantly enhances innovative behavior (β = 0.338, *p* < 0.001) and positively influences role width self-efficacy (β = 0.530, *p* < 0.001). Furthermore, when role width self-efficacy was introduced as a mediating variable, it significantly impacted innovative behavior (β = 0.375, *p* < 0.001).

Wu and Zhao (2010) investigated the influence of leadership style on employees' innovative behavior and found that transactional leadership, when paired with goal-oriented actions, can stimulate employees' innovative potential. Wang et al. (2014) identified a significant positive link between transactional leadership and employees' self-efficacy, influencing not only their perception of their leaders but also their responses to them. Lu et al. (2006) also observed a positive correlation between self-efficacy and employees' innovative behaviors. Bass and Riggio (2006) drawing on Maslow's Hierarchy of Needs Theory, suggest that transactional leadership traits can satisfy the fundamental needs of employees, which in turn shape their values and attitudes toward their work. In light of these considerations, the following hypotheses are proposed:

Hypothesis 7: perceived organizational support mediates the relationship between ambidextrous leadership and knowledge worker innovation behavior.

Hypothesis 8: perceived organizational support mediates the relationship between ambidextrous leadership and employees' general self-efficacy.
Hypothesis 9: general self-efficacy mediates between ambidextrous leadership and knowledge worker innovation behavior.
Hypothesis 10: general self-efficacy mediates between Perceived organizational support and knowledge worker innovation behavior.

While existing studies do not entirely align with the complete chain mediation model linking ambidextrous leadership to perceived organizational value, self-efficacy, and employee behavior, Deng (2021) demonstrates that ambidextrous leadership to integrating transformational and transactional styles to positively influences proactive change behavior. Xu (2023) further asserts that ambidextrous leadership enhances improvisational behavior by boosting employees' organizational self-esteem, or their perceived value within the organization. Additionally, Zhang and Zhao (2024) identifies that innovative self-efficacy positively moderates the relationship between organizational harmony and innovative behavior. Although his model uses inclusive leadership as the independent variable, its pathway from organizational harmony to self-efficacy offers insights for ambidextrous leadership research. This suggests that perceived organizational value and self-efficacy may form a chain mediation. In essence, ambidextrous leadership indirectly boosts employees' self-efficacy by enhancing their perception of organizational value which subsequently influences specific behaviors. In view of this, the following hypothesis is proposed.

Hypothesis 11: perceived organizational support and employees' general self-efficacy play a chain mediating role in the relationship between ambidextrous leadership and knowledge worker innovation behavior.

The interplay between transformational and transactional leadership is inherently non-linear; their combined efficacy oscillates between synergistic enhancement and antagonistic interference, contingent on implementation intensity and contextual boundaries. The present study posit that ambidextrous leadership operates through thresholded complementarity. In this model, transformational elements dominate in early innovation phases to ignite ideation, while transactional components gain primacy during implementation to ensure discipline. This dynamic equilibrium defies simple linear aggregation, necessitating methodological sophistication to capture phase-specific contingencies.

While existing research confirms the beneficial effects of ambidextrous leadership on innovation, three significant gaps remain in the literature. First, current studies predominantly examine a single mediation pathway and have not validated the chain mediation involving organizational support and self-efficacy. Second, the evidence is largely based on Western samples, leaving the mediating effects of high power distance cultures, such as in China, unexplored. Third, traditional regression models struggle to capture non-linear relationships, and the application of hybrid SEM-ANN methods remains unaddressed.

**Figure 2:**
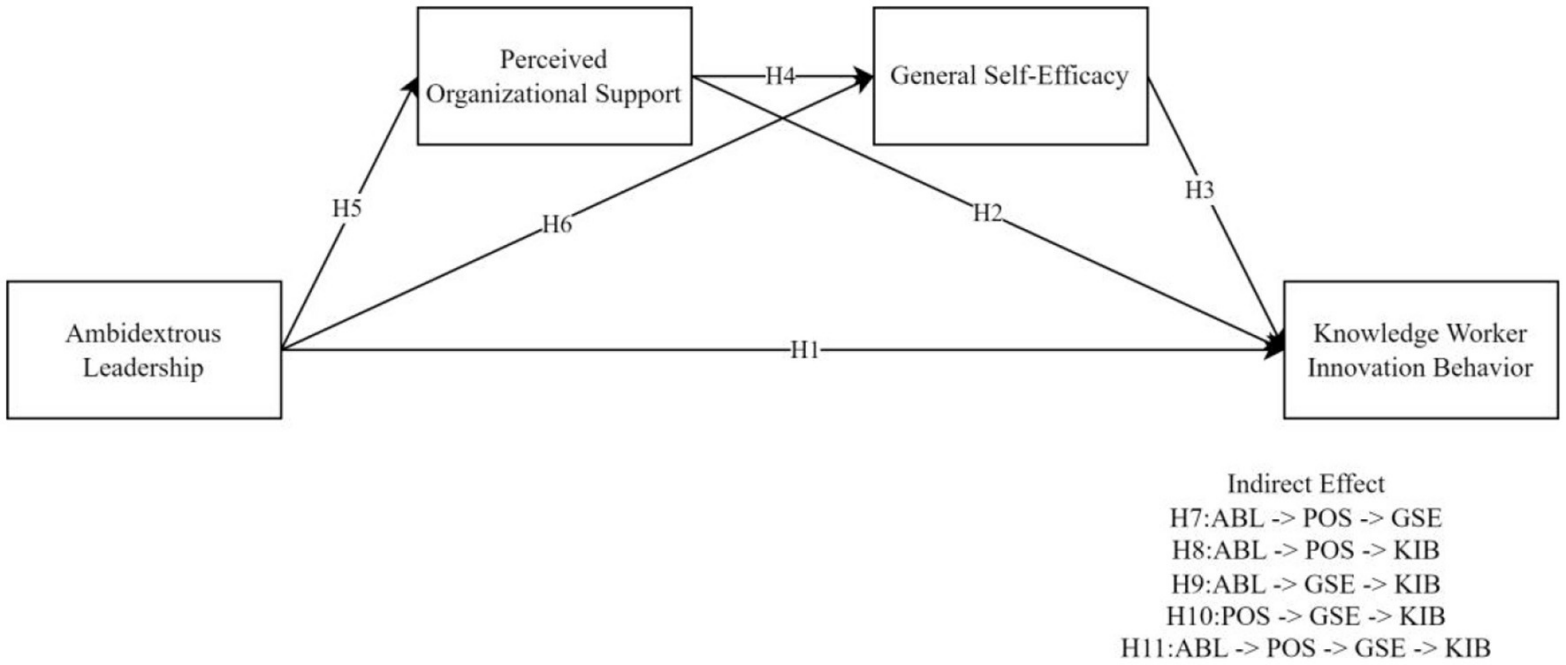
Theoretical framework.

Based on the above analysis and the proposed research hypotheses, this study established a conceptual model of the relationship between ambidextrous leadership (ABL), perceived organizational support (POS), general self-efficacy (GSE), and knowledge worker innovation behavior (KIB), as illustrated in Figure 2.

## 3 Methodology

### 3.1 Research sample and procedures

The sample size is generally determined by the number of variables observed. For normally distributed data, Bentler and Chou (1987) advise a minimum of 5 cases per variable when multiple indicators of the latent variable are present. A more common guideline by Nunnally et al. (1967) suggests at least 10 cases per indicator as the minimum sample size. Our study involves four variables with a total of 27 indicators, according to Nunnally et al. (1967) the minimum required sample size is 270. As the survey was conducted online, the actual sample size is expected to surpass this threshold due to the broad participant diversity and coverage.

In this study, a random sampling method was employed to disseminate online questionnaires to knowledge workers across more than 100 cities and diverse industries in China via CREDAMO between January and June 2024. A total of 400 questionnaires were distributed, yielding a remarkable response rate of 96%, with 385 questionnaires ultimately retrieved. The recovered questionnaires underwent a screening process, excluding those with response times below the average, resulting in the removal of 28 questionnaires and leaving 372 valid data points, which represents 93% of the original sample. Table 1 shows the profile of respondents.

**Table 1:** Demographic profile of respondents (*N* = 372).

**Characteristics of respondents**	**Classification**	**Sample amounts**	**Percentage (%)**
Gender	Male	99	26.60%
	Female	273	73.40%
Age Group	21–30	89	23.90%
	31–40	248	66.70%
	41–50	22	5.90%
	51–60	13	3.50%
Educaiton	Master degree	345	92.70%
	PhD	27	7.30%
Occupation	State-owned enterprises	71	19.08%
	Institutions	36	9.67%
	Civil service	23	6.18%
	Private enterprises	210	56.45%
	Foreign-funded enterprises	32	8.62%
Position	Ordinary employees	91	24.50%
	Lower management position	66	17.80%
	Middle management position	130	34.90%
	Senior management position	85	22.80%

### 3.2 Measurement

In this research, we employed a five-point Likert scale to evaluate four key dimensions: ambidextrous leadership, perceived organizational support, general self-efficacy, and the innovative behaviors of knowledge workers. For each item in the questionnaire, responses were collected on a 5-point Likert scale, where 1 show 'strongly disagree' and 5 indicates “strongly agree”.

Ambidextrous leadership (ABL) was assessed using a scale developed by Han et al. (2016) which was refined based on employee feedback. This scale initially comprised seven items for transformational leadership and five for transactional leadership. Following factor analysis, five items were removed due to low loading, resulting in a final count of seven reliable items. An example item is: “Leaders demonstrate resolve in achieving objectives.” The scores for ambidextrous leadership were calculated by averaging the scores of both transformational and transactional leadership dimensions, yielding a scale reliability of 0.76.

Perceived organizational support (POS) was measured using a scale by Eisenberger (Eisenberger et al., 1986) as rated by employees. The initial scale consisted of eight items, of which two were removed after factor analysis due to low loading, leaving six items. An example item is: “The organization considers my suggestions.” This scale demonstrated an internal consistency of 0.874.

General self-efficacy (GSE) was assessed using the English version of the scale developed by Schwarzer and Jerusalem (1995) translated into Chinese by Wang et al. (2001). Of the ten items initially presented, three were removed due to low factor loading, resulting in seven valid items. A sample item is: “I am capable of obtaining what I desire even in the face of opposition.” The internal consistency of this scale was 0.870.

Knowledge worker innovation behavior (KIB) was evaluated using a scale developed by Zhang et al. (2016), which initially included eight items rated by employees. After factor analysis, one item with low loading was removed, resulting in seven effective items. An example item is: “I frequently seek opportunities to enhance my work methods and processes.” The scale's internal consistency was 0.838.

### 3.3 Data analysis and procedure

In our quest to evaluate the efficacy of the model, we employed the Partial Least Squares (PLS) method, a sophisticated approach within the framework of Structural Equation Modeling (SEM). This method is particularly advantageous as it seamlessly integrates the processes of measurement and structural modeling (Bollen, 2014). In this context, the measurement model is responsible for examining the theoretical connections between observed indicators and their underlying latent constructs. Conversely, the structural model quantifies the hypothesized relationships that exist between exogenous variables, which are independent in nature, and endogenous variables, which depend on other factors within the model.

## 4 Data analysis and result

In light of the exploratory and predictive nature of this research, we have selected the Partial Least Squares Structural Equation Modeling (PLS-SEM) approach to enhance the accuracy of predictions. This method is particularly well-suited for evaluating causal predictive relationships, which are essential for the development of models and theories. As illustrated in Figure 1, the analysis of the models within this study was conducted using SmartPLS 4.0 for both the measurement and structural models. The study features a reflective measurement model, prior to further analysis, a descriptive analysis of the data collected in this survey was conducted.

### 4.1 Descriptive analysis

The descriptive statistical results for the main variables, along with the correlation coefficient matrix, are presented in Table 2. The findings indicate a significant correlation between ambidextrous leadership (ABL) and three key variables: perceived Organizational Support (POS), General Self-Efficacy (GSE), and Knowledge Worker Innovative Behavior (KIB). These results provide preliminary evidence that supports the subsequent verification of the hypotheses.

**Table 2:** Descriptive analysis and correlation.

**Variable**	**Mean**	**STD**	**1**	**2**	**3**	**4**	**5**	**6**	**7**	**8**
1. Gender	1.73	0.44								
2. Age	1.89	0.65	0.010							
3. Degree	1.07	0.26	−0.043	0.111^*^						
4. Occupation	3.27	1.32	0.136^**^	0.194^**^	−0.088					
5. Duty	2.56	1.09	0.159^**^	0.426^**^	−0.011	0.309^**^				
6. POS	3.95	0.69	0.006	0.223^**^	−0.052	0.177^**^	0.376^**^			
7. GSE	3.87	0.66	−0.002	0.270^**^	−0.047	0.183^**^	0.370^**^	0.659^**^		
8. KIB	4.00	0.62	0.075	0.250^**^	−0.040	0.210^**^	0.435^**^	0.745^**^	0.765^**^	
9. ABL	16.96	4.03	0.055	0.255^**^	−0.034	0.215^**^	0.442^**^	0.742^**^	0.641^**^	0.707^**^

^*^Significant at the 5% level; ^**^Significant at the 1% level.

### 4.2 Stage-1: PLS-SEM analysis

#### 4.2.1 Reflective measurement model assessment

Reflective measurement model evaluation is commonly used in PLS-SEM, particularly when assessing latent variables. Its primary function is to validate and assess the validity and reliability of the reflective measurement model. The results presented in Table 3 indicate that the first indicator for the ambidextrous leadership (ABL) construct exhibited a loading value slightly below the ideal threshold of 0.60. Although the AVE was marginally below the 0.50 benchmark, it is noteworthy that a value of 0.40 is still considered acceptable (Lam, 2012). This aligns with the assertion made by Fornell and Larcker (1981) that a construct maintains adequate convergent validity if its AVE is less than 0.50 but its composite reliability exceeds 0.60. The factor loading of ABL1 falls below the recommended threshold of 0.6.However, Field (2024) suggests that a factor can be considered reliable if it exhibits four or more loadings of at least 0.6, irrespective of sample size. Therefore, ABL1 can be retained despite its suboptimal individual loading.

**Table 3:** Result summary for reflective model.

	**Indicators**	**Collinearity statistics**	**Convergent validity**		**Internal consistency reliability**		**Discriminant validity**
		**VIF**<**5**	**Loading**	**AVE**	**Cronbach's Alpha (CA)**	**CR**	**HTMT**<**0.85 (0.9)**
ABL	ABL1	1.224	0.53	0.416	0.764	0.775	POS (0.893) GSE (0.751) KIB (0.857)
	ABL2	1.543	0.747				
	ABL3	1.347	0.647				
	ABL4	1.328	0.623				
	ABL5	1.360	0.660				
	ABL6	1.291	0.617				
	ABL7	1.381	0.668				
POS	POS1	1.914	0.794	0.617	0.876	0.879	ABL (0.893) GSE (0.745) KIB (0.858)
	POS2	1.912	0.790				
	POS3	1.913	0.790				
	POS4	1.673	0.730				
	POS5	1.858	0.775				
	POS6	2.172	0.831				
GSE	GSE1	1.593	0.710	0.565	0.87	0.874	ABL (0.751) POS (0.745) KIB (0.887)
	GSE2	1.989	0.783				
	GSE3	2.197	0.807				
	GSE4	1.899	0.771				
	GSE5	1.503	0.671				
	GSE6	1.452	0.671				
	GSE7	2.431	0.833				
KIB	KIB1	1.418	0.6	0.513	0.841	0.846	ABL (0.857) POS (0.858) GSE (0.887)
	KIB2	1.468	0.667				
	KIB3	1.417	0.657				
	KIB4	1.928	0.790				
	KIB5	1.737	0.758				
	KIB6	1.677	0.747				
	KIB7	1.632	0.720				

In light of these standards, the ABL construct is deemed to exhibit satisfactory internal consistency and discriminant validity. The remaining reflective constructs have also been found to meet the established criteria for reliability and validity. Additionally, the HTMT values, all of which are below the critical threshold of 0.90, confirm the discriminant validity of the constructs examined in this study.

#### 4.2.2 Structure model assessment

**Figure 3:**
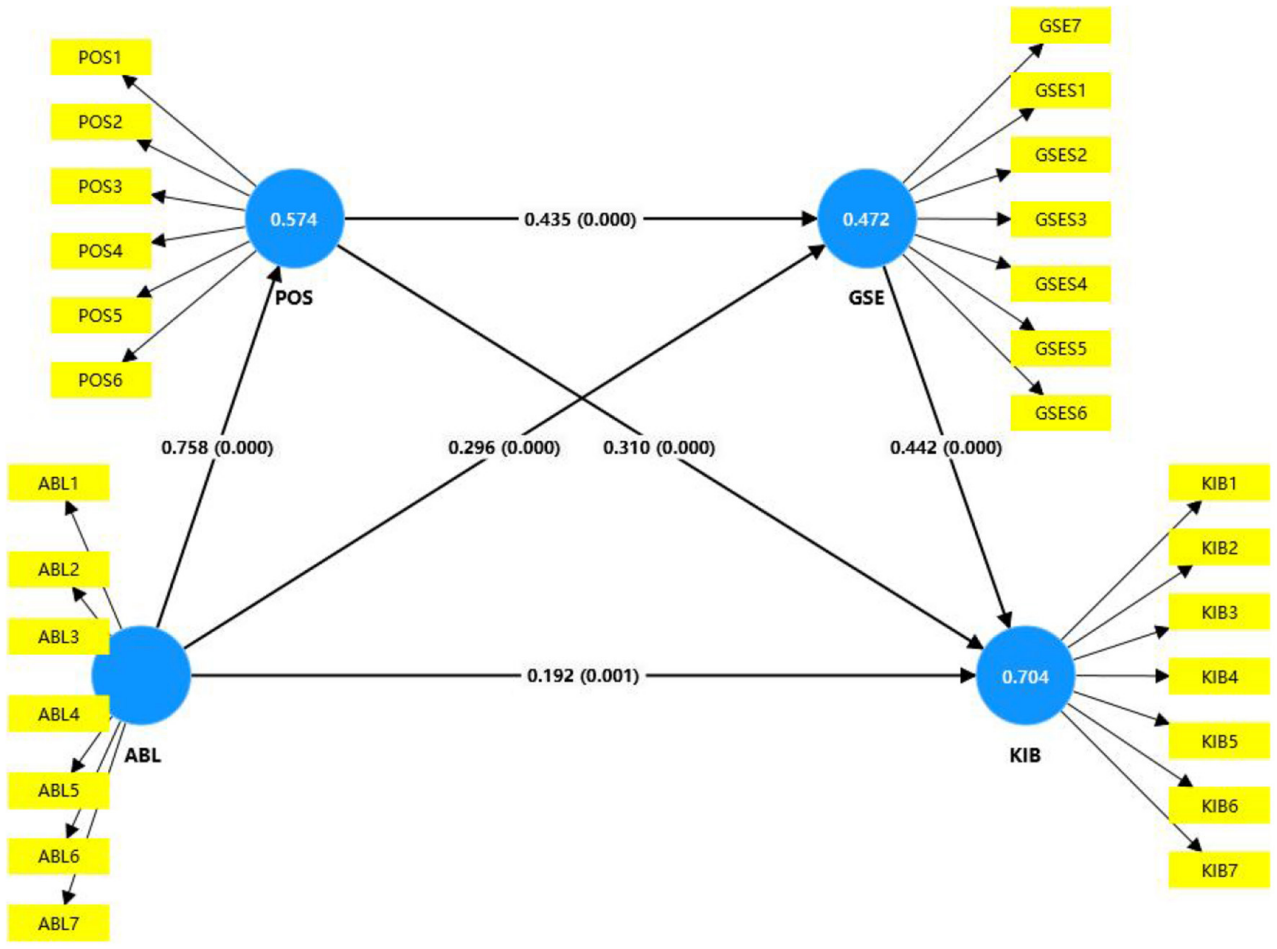
Structure model result.

Structural model evaluation is a critical step in SEM analysis, used to assess the relationships between latent variables and their path model. Unlike measurement model evaluation, structural model evaluation primarily focuses on determining whether the causal relationships between latent variables hold and whether the model can effectively predict or explain the observed data. Table 4 and Figure 3 illustrate the model's explanatory power, with R^2^ values of 0.704, 0.574, and 0.472, respectively. These values exceed the established criteria, indicating that the model is robust for the purposes of this study. As detailed in Table 4, the direct effects confirm the significance of hypotheses H1 to H6, with *p*-values significantly below the 0.05 threshold. Table 5 elucidates the indirect effects, revealing significant mediation effects for hypotheses H7 to H11. These findings indicate that perceived organizational support acts as a mediator in the relationship between ambidextrous leadership, general self-efficacy, and knowledge workers' innovative behavior. Moreover, general self-efficacy also plays a mediating role in the relationship between ambidextrous leadership and knowledge workers' innovative behavior. Furthermore, perceived organizational support and general self-efficacy are identified as sequential mediators in the relationship between ambidextrous leadership and knowledge workers' innovative behavior, illustrating a chain mediation dynamic.

**Table 4:** Significance testing result of the structure model path coefficient.

**Relationship**	**Path coefficients**	***t* value**	***p* value**	**Significance (*p* < 0.05)?**	***R* ^2^**	***Q* ^2^**	***f* ^2^**
H1: ABL -> KIB	0.192	3.460	0.001	Yes	0.704	0.354	0.049
H2: POS -> KIB	0.310	5.677	0.000	Yes			0.120
H3: GSE -> KIB	0.442	9.164	0.000	Yes			0.349
H4: POS -> GSE	0.435	6.509	0.000	Yes			0.153
H5: ABL -> POS	0.758	26.382	0.000	Yes	0.574	0.348	1.347
H6: ABL -> GSE	0.296	4.083	0.000	Yes	0.472	0.264	0.071

**Table 5:** Significance analysis of the indirect effects.

**Relationship**	**Path coefficients**	***t* value**	***p* value**	**Significance (*p* < 0.05)?**
H7: ABL -> POS -> GSE	0.330	6.277	0.000	Yes
H8: ABL -> POS -> KIB	0.235	5.593	0.000	Yes
H9: ABL -> GSE -> KIB	0.131	3.738	0.000	Yes
H10: POS -> GSE -> KIB	0.192	5.406	0.000	Yes
H11: ABL -> POS -> GSE -> KIB	0.146	5.257	0.000	Yes

Table 6 presents the comparative PLS predict outcomes of this study. Although not all PLS-SEM values exceed the LM benchmarks, following the guidance of Sarstedt et al. (2021), the majority of PLS-SEM values being lower than those of the LM indicates that the model demonstrates a medium level of predictive capability.

**Table 6:** PLS_predict_ result.

	**Q^2^predict**	**PLS-SEM_RMSE**	**PLS-SEM_MAE**	**LM_RMSE**	**LM_MAE**
GSES1	0.197	0.785	0.591	0.790	0.594
GSES2	0.246	0.921	0.750	0.914	0.727
GSES3	0.231	0.784	0.595	0.792	0.603
GSES4	0.200	0.748	0.560	0.759	0.570
GSES5	0.175	0.619	0.507	0.627	0.513
GSES6	0.184	0.724	0.588	0.733	0.595
GSES7	0.257	0.837	0.661	0.838	0.666
KIB1	0.192	0.633	0.519	0.639	0.518
KIB2	0.201	0.675	0.555	0.679	0.551
KIB3	0.145	0.712	0.552	0.726	0.553
KIB4	0.352	0.831	0.667	0.824	0.660
KIB5	0.333	0.893	0.696	0.902	0.704
KIB6	0.284	0.770	0.596	0.780	0.609
KIB7	0.202	0.640	0.521	0.646	0.520
POS1	0.389	0.630	0.510	0.631	0.513
POS2	0.343	0.800	0.612	0.801	0.617
POS3	0.354	0.744	0.603	0.753	0.607
POS4	0.233	0.664	0.515	0.667	0.516
POS5	0.345	0.670	0.548	0.670	0.546
POS6	0.418	0.713	0.545	0.709	0.544

### 4.3 Stage-2: artificial neural network (ANN)

While SEM effectively explains multivariate linear relationships between variables, its predictive power may be limited when addressing non-linear and non-compensatory relationships. Thus, although SEM models simplify the decision-making process, they may still have limitations in capturing complex relationships.

To address these limitations, this study further employs the multi-layer perceptron (MLP) approach in neural networks, constructing artificial neural network models based on the SEM hypothesis testing results. The multi-layer perceptron (MLP) is a neural network model designed to handle complex non-linear relationships. By employing the artificial neural network (ANN) model, we can more accurately capture and predict non-linear relationships in the data, thereby enhancing predictive accuracy and providing more robust support for decision-making.

**Figure 4:**
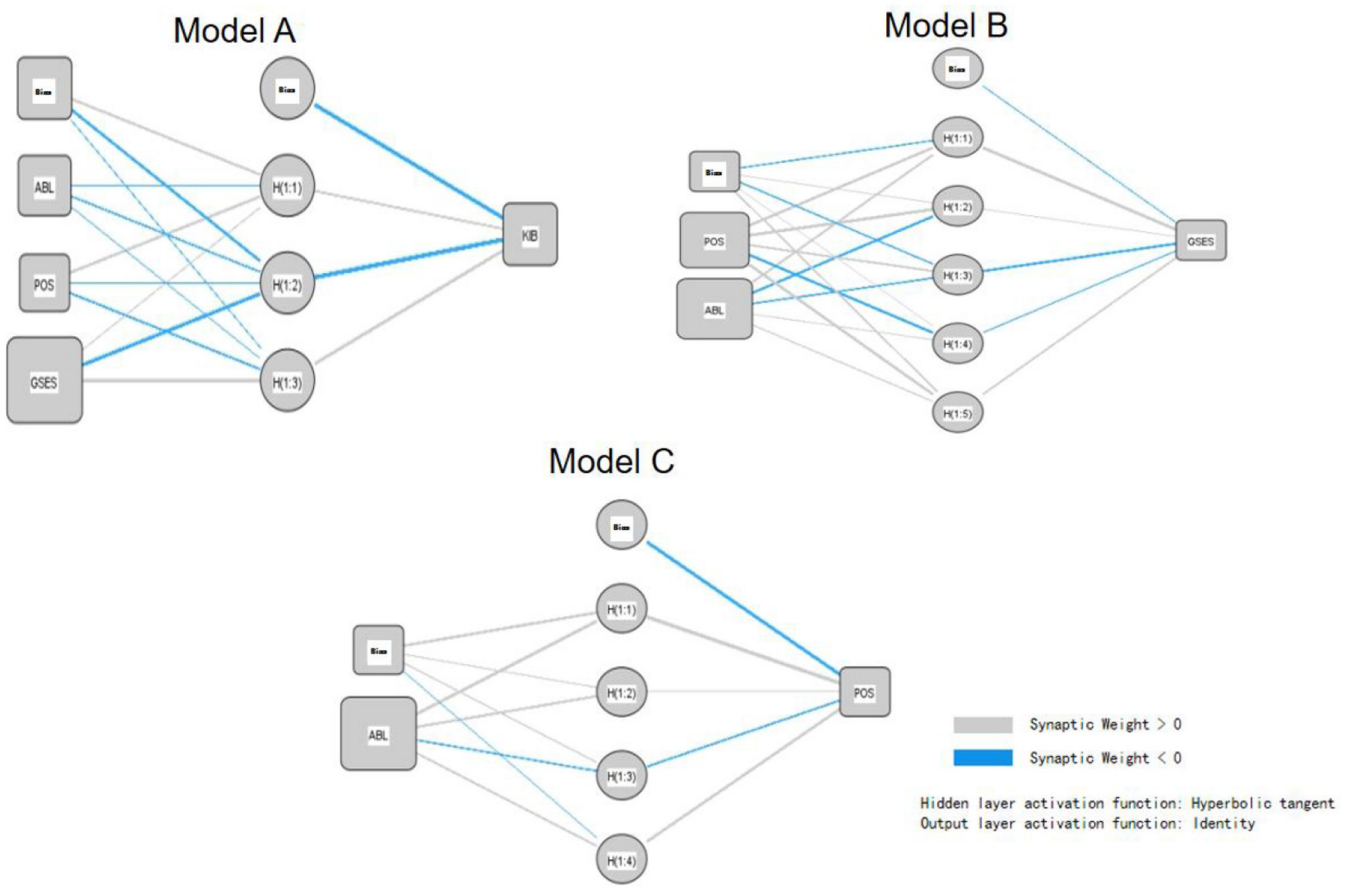
ANN diagram.

Based on the SEM results shown above, we decomposed the research model into three ANN models, each corresponding to one of the three dependent variables (KIB, GSE, POS), which were identified as the output layer. Significant predictors from the SEM were then used as inputs for these three models as shown in Figure 4.

To evaluate the predictive performance of the ANN models A, B, and C, we employed ten-fold cross-validation. During this process, the data set was divided into ten equal parts, with 90% of the data randomly selected for training and the remaining 10% used for testing in each iteration. This process was repeated ten times, ensuring each part served as the test set once to assess the models' performance.

Model A used KIB as the dependent variable and included absorptive capacity (ABL), POS, and GSES as covariates. It employed an ANN with a single hidden layer containing three units. As noted in Abiodun et al. (2018), a single hidden layer effectively balances computational efficiency and non-linear fitting for medium-complexity problems. The model's covariate-to-hidden-unit ratio (1:1) aligns with the minimum guideline (1:2–1:5) from Justino et al. (2024), ensuring overfitting is minimized while preserving the ability to analyze the interaction effects of ABL, POS, and GSE on KIB.

Model B utilized GSE as the dependent variable and ABL, POS as the covariate, employing an ANN with a single hidden layer. This layer comprised five computational units, exceeding the number of covariates 2 to effectively capture the non-linear dynamic relationship between ABL and POS. As noted in Abiodun et al. (2018) neural networks require additional hidden units to enhance feature expression in time-series interactions, aligning with this design rationale.

Model C employed POS as the dependent variable and ABL as the sole covariate in an ANN model with one hidden layer comprising 4 units. This mirrors the single-covariate structure of the medical diagnosis model in Abiodun et al. (2018). The 4 hidden units effectively capture the non-linear threshold effect of ABL on POS. Justino et al. (2024) highlights that in such a model, the number of hidden units should align with the data's non-linear strength. If the ABL to POS relationship exhibits high-order non-linearity, the 4 unit structure offers adequate fitting flexibility; otherwise, excess parameters may increase the error rate.

Using this validation method, we calculated the root mean square error (RMSE) for each ANN model. As shown in Table 7, the RMSE values ranged from 0.143 to 0.244 these values indicate that the prediction errors of the models are relatively small, demonstrating that the ANN models produced accurate predictions on the test data. This provides reliable support for further analysis and decision-making.

**Table 7:** RMSE validation of ANN models.

	**Model A**		**Model B**		**Model C**	
	**Input: ABL, POS, GES**		**Input: POS, ABL**		**Input: ABL**	
	**Output: KIB**		**Output: GSE**		**Output: POS**	
Neural network	Training	Testing	Training	Testing	Training	Testing
ANN1	0.141	0.214	0.246	0.227	0.230	0.112
ANN2	0.165	0.082	0.248	0.169	0.221	0.209
ANN3	0.150	0.120	0.248	0.121	0.207	0.226
ANN4	0.154	0.133	0.240	0.180	0.236	0.244
ANN5	0.149	0.204	0.260	0.205	0.209	0.222
ANN6	0.157	0.196	0.241	0.175	0.220	0.206
ANN7	0.183	0.117	0.245	0.269	0.209	0.258
ANN8	0.182	0.146	0.240	0.260	0.214	0.252
ANN9	0.152	0.135	0.240	0.264	0.228	0.076
ANN10	0.149	0.081	0.236	0.300	0.235	0.179
Mean	0.158	0.143	0.244	0.217	0.221	0.198
SD	0.119	0.218	0.082	0.237	0.104	0.246

As shown in Table 8, sensitivity analysis reveals the normalized importance ranking of covariates for the dependent variables in each ANN model. Table 8 reveals that the average RMSE for the ANN test set is notably lower than that of the training set, demonstrating the model's robust generalization capability and its proficiency in capturing non-linear relationships. In Model A, the importance ranking of predictors is as follows: GSE (100%), POS (69.085%), ABL (61.322%). In Model B, the predictor importance ranking is: ABL (100%), POS (96.348%). In Model C, the importance of the ABL predictor is 100%. Model A's findings indicate a non-linear superposition effect, with self-efficacy exerting the greatest influence followed by perceived organizational support (69.1%) and ambidextrous leadership behavior (61.3%). The varying magnitudes of these variables suggest a complex non-linear relationship. Model B demonstrates that ambidextrous leadership behavior outweighs perceived organizational support in importance, suggesting it predominantly influences changes in employees' self-efficacy through non-linear pathways at high intensities. This difference highlights the sensitivity of different models to variable importance and helps us understand which variables have a more significant impact on the dependent variable in each model.

**Table 8:** Normalized importance analysis in ANN models.

**Neural network**	**Model A (Output: KIB)**			**Model B (Output: GSE)**		**Model C (Output: POS)**
	**ABL**	**POS**	**GSE**	**POS**	**ABL**	**ABL**
ANN1	0.271	0.306	0.422	0.466	0.534	1.000
ANN2	0.218	0.269	0.512	0.542	0.458	1.000
ANN3	0.238	0.312	0.450	0.450	0.550	1.000
ANN4	0.250	0.215	0.535	0.512	0.488	1.000
ANN5	0.322	0.258	0.419	0.510	0.490	1.000
ANN6	0.474	0.200	0.326	0.464	0.536	1.000
ANN7	0.172	0.418	0.416	0.493	0.507	1.000
ANN8	0.225	0.369	0.405	0.409	0.591	1.000
ANN9	0.240	0.328	0.432	0.545	0.455	1.000
ANN10	0.252	0.324	0.424	0.516	0.484	1.000
Average relative importance	0.266	0.300	0.434	0.491	0.509	1.000
Normalized relative importance (%)	61.322	69.085	100.000	96.348	100.000	100.000

The study conducted a priority ranking analysis by comparing the path coefficients of the SEM and the normalized relative importance rankings of the ANN (as shown in Table 9). The comparison results indicate that the ranking outcomes of PLS-SEM-ANN models for Model A and Model C are entirely consistent. This suggests that the relative importance rankings of variables remain unchanged regardless of whether evaluated using SEM or ANN. For Model B, some paths exhibited slight inconsistencies. Overall, the SEM-ANN models in this study demonstrate strong explanatory power, providing a solid foundation for model reliability and theoretical support.

**Table 9:** Comparative analysis of the SEM-ANN results.

**SEM path**	**SEM: path coefficient**	**ANN: normalized relative importance (%)**	**SEM ranking**	**ANN ranking**	**Remark**
**Model A (Output: KIB)**					
ABL	0.192	61.322	3	3	Match
POS	0.310	69.085	2	2	Match
GSE	0.442	100.000	1	1	Match
**Model B (Output: GSE)**					
POS	0.435	96.348	1	2	Unmatched
ABL	0.296	100.000	2	1	Unmatched
**Model C (Output: POS)**					
ABL	0.758	100.000	1	1	Match

Comparative analysis of SEM and ANN reveals that in the linear stage, low ABL intensity is primarily influenced by ABL → POS → KIB. In the non-linear stage, with high ABL intensity, ANN highlights threshold effects, such as employees' self-efficacy, and regulatory reversals, like the saturation effect of perceived organizational support as key mechanisms.

## 5 Discussion and implications

This study examines the direct impact of ambidextrous leadership on the innovative activities of knowledge workers within the distinctive cultural context of China. Furthermore, based on the theory of triple interaction, it posits that ambidextrous leadership may indirectly stimulate employees' innovative behavior by enhancing their self-efficacy. Additionally, perceived organizational support is suggested to reinforce this influence. The study evaluates how ambidextrous leadership—encompassing both transformational and transactional styles—positively influences the innovative behavior of knowledge workers through perceived organizational support and general self-efficacy, employing a PLS structural equation model for this analysis.

Our research aligns with the principles of reciprocal determinism theory, which explains the complex interactions between individuals, their behaviors, and the broader context. The results indicate that ambidextrous leadership significantly enhances employees' self-efficacy, which, in turn, indirectly stimulates their innovative behaviors. In this transformation process, organizational support emerges as a key mediating factor. This finding supports reciprocal determinism theory, which posits that environmental factors play a decisive role in shaping individual behavior. It also provides new empirical evidence for triadic interaction theory, demonstrating that dual leadership affects employees' self-efficacy and, consequently, their propensity to innovate. This strengthens the theory's assertion about the critical influence of environmental factors on individual behavior, thus enriching existing research on organizational behavior. Additionally, our study explores the role of perceived organizational support within the framework of reciprocal determinism theory from a novel perspective. Traditionally, perceived organizational support has been regarded solely as an environmental factor, but our study reveals that it exerts a more nuanced influence on the relationship between leadership behaviors and employee innovation, suggesting the presence of more complex underlying psychological mechanisms. In summary, this study elucidates the mechanisms through which leadership behaviors and perceived organizational support influence employee innovation through mutual determinism. By highlighting the synergistic impact of dual leadership, perceived support, and self-efficacy on knowledge worker innovation, this study offers unique insights into strategically nurturing employee behaviors through leadership and cultural dynamics.

The findings align with previous research that has investigated the influence of ambidextrous leadership on employee behavior and organizational outcomes. This leadership approach combines the traits of transformational and transactional leadership to achieve a balance between innovation and efficiency. Regarding innovative behavior, numerous studies have focused on how ambidextrous leadership encourages employees to engage in innovative work behavior, directly investigated in this study (Dinesh Babu et al., 2024). It is evident that mediating variables play a pivotal role in this context; perceived organizational support and general self-efficacy have been identified as mediators influencing the relationship between ambidextrous leadership and employee innovative behavior. In a similar vein, Jia et al. (2024) and Cheng (2024) propose the inclusion of additional mediating variables, including cognitive load, emotional exhaustion, innovative self-efficacy, and harmonious work enthusiasm.

While the majority of studies adopt ambidextrous leadership as their central theme, there is significant variation in their specific research focus. For instance, Jia et al. (2024) investigate the potential adverse effects of ambidextrous leadership on the well-being of managers, while our research aims to examine how ambidextrous leadership influences the innovative behavior of knowledge workers through various mediating variables. In terms of industry context, while Dinesh Babu et al. (2024) focus on the IT sector to assess how ambidextrous leadership shapes innovative work behaviors and employee performance, our study explores the broader applicability to knowledge workers. Additionally, while most studies employ quantitative techniques such as structural equation modeling, Malik et al. (2024) utilize qualitative case studies to examine the impact of ambidextrous leadership on human resource management practices and strategic agility, highlighting the diversity of research methods in this area.

Regarding cultural and geographical contexts, our study concentrate on organizational behavior within a Chinese cultural framework, whereas other studies may cover various cultural environments. Gouda and Tiwari (2024) stand out for their comprehensive exploration of the Coronavirus pandemic's impact on the work environment and the pivotal role of ambidextrous leadership in fostering agile mindsets among employees. Additionally, Paper 4 introduces the concept of “Zhong Yong thinking” as a moderating variable, aiming to integrate traditional Chinese cultural concepts with modern management theory, although other studies may adopt different theoretical frameworks. Nasution et al. (2024) provide a detailed analysis of the impact of ambidextrous leadership, talent management, and individual ambidexterity on pharmaceutical representatives' performance, considering work engagement's mediating effect and offering insights for the pharmaceutical industry, in contrast to our focus on theoretical contributions.

The complex and multifaceted nature of ambidextrous leadership theory is exemplified by the enhancement of theoretical frameworks, industry-specific nuances in leadership application, a diverse range of research methodologies, and considerations of cultural diversity. Mueller et al. (2020) emphasize the importance of integrating interactions among different organizational levels within theoretical models. In the context of the public museum sector, Kung et al. (2020) highlight the broad applicability and universal relevance of ambidextrous leadership concepts. In contrast to the cross-sectional surveys typical of other studies, Gerlach et al. (2020) employ a longitudinal research approach, offering a dynamic perspective on the influence of ambidextrous leadership and illustrating the critical nature of research design selection. While the impact of cultural differences on the efficacy of ambidextrous leadership remains underexplored in existing literature, identifying research gaps and future trajectories presents novel opportunities for inquiry in both theoretical and practical domains.

The empirical depth of research, the spectrum of leadership behaviors, and their adaptability further enrich the ambidextrous leadership framework. Rosing and Zacher (2023) and Gerlach et al. (2020) provide tangible insights into how ambidextrous leadership behaviors can influence innovation performance, aligning with the theoretical postulations presented in this study. Ouyang et al. (2022) explore the dynamics of combining transformational and transactional leadership and their effects on employee voice through the lens of work motivation. Busola Oluwafemi et al. (2020) reveal the intermediary role of adaptive leadership behaviors in the relationship between ambidextrous leadership and employee innovation, emphasizing leaders' capacity to adapt their approaches in varying contexts to stimulate innovation. These unexpected findings yield new theoretical insights related to ambidextrous leadership, delineating prospective trajectories for future scholarly inquiry and practical application.

## 6 Limitation and future work

This study elucidates the influence of ambidextrous leadership on the innovative behavior of knowledge workers, while also identifying several limitations. The sample size may be confined to specific sectors or geographical locations, potentially skewing the generalizability of the findings. Furthermore, the study's design may not fully capture the evolving dynamics between ambidextrous leadership and innovative employee behavior, underscoring the need for longitudinal research to address this gap. Although the measurement instruments were selected with care, their psychometric properties require further validation across diverse cultural and organizational settings. Additionally, other significant mediating and moderating factors, such as organizational culture and market pressures, may have influenced the results. Finally, while our work is grounded in established theoretical frameworks, there remains an opportunity for further research to extend our theoretical scope.

Moreover, employing experimental methodologies could allow for a more precise assessment of causality, particularly regarding the impact of ambidextrous leadership training interventions. It would be prudent and necessary to further develop ambidextrous leadership theory, informed by our findings and tailored to various organizational contexts. Translating the ambidextrous leadership concept into practical organizational strategies and metrics for implementation success will significantly benefit the practitioner community. Finally, future studies might explore the potential impact of technological advancements on ambidextrous leadership, as the integration of technology could serve as a catalyst for ambidextrous leadership, influencing employees' innovative behavior in novel and transformative ways.

## Data Availability

The datasets presented in this study can be found in online repositories. The names of the repository/repositories and accession number(s) can be found at: https://doi.org/10.3886/E209282V1.
